# B‐type natriuretic peptide is associated with remodeling and exercise capacity after transcatheter aortic valve replacement for aortic stenosis

**DOI:** 10.1002/clc.23138

**Published:** 2018-12-31

**Authors:** Kimi Sato, Arnav Kumar, Amar Krishnaswamy, Stephanie Mick, Milind Y. Desai, Brian P. Griffin, Samir R. Kapadia, Zoran B. Popović

**Affiliations:** ^1^ Department of Cardiovascular Medicine Heart and Vascular Institute, Cleveland Clinic Cleveland Ohio

**Keywords:** aortic stenosis, B‐type natriuretic peptide, left ventricular remodeling, transcatheter aortic valve replacement

## Abstract

**Background:**

We aimed to assess longitudinal changes of B‐type natriuretic peptide (BNP) in aortic stenosis (AS) patients treated by transcatheter aortic valve replacement (TAVR).

**Methods:**

From our TAVR database, we identified 193 consecutive patients with severe symptomatic AS who underwent TAVR and were prospectively followed using serial BNP levels and echocardiography. Patients were divided into subgroups according to type of left ventricular (LV) remodeling as having normal LV mass and relative wall thickness, or showing concentric remodeling (CR), concentric hypertrophy (CH), and eccentric hypertrophy (EH).

**Results:**

At baseline, 30 patients (16%) had EH, 115 (60%) had CH, 37 (19%) had CR, and 11 (6%) had normal LV geometry. After TAVR, BNP decreased in the first 30 days, with further improvement during follow‐up. Patients with EH had higher BNP at baseline (*P* < 0.01) and a greater subsequent decrease (*P* < 0.001). During the median follow‐up of 1331 days (interquartile range: 632‐1678), 119 (62%) patients died. BNP showed a time‐dependent association with all‐cause mortality both in a univariable (hazards ratio [HR] 1.24, 95% confidence interval [CI]: 1.04‐1.47, *P* = 0.017), and in a multivariable model with Society of Thoracic Surgeons score and baseline BNP forced into the analysis (HR 1.32, 95% CI: 1.001‐1.73, *P* = 0.049). Elevated BNP was associated with a larger LV end‐diastolic volume index (*P* < 0.001) and shorter 6‐minute walk test distance (*P* = 0.013) throughout follow‐up.

**Conclusion:**

In patients with AS, BNP was associated with LV remodeling phenotypes and functional status before and after TAVR. Elevated BNP levels were associated with poor prognosis.

## 1 INTRODUCTION

B‐type natriuretic peptide (BNP) levels are associated with disease severity, left ventricular (LV) remodeling, and prognosis in severe aortic stenosis (AS).^1^, ^2^ While studies have shown the prognostic utility of BNP at baseline and early after transcatheter aortic valve replacement (TAVR) in severe AS patients who underwent TAVR,^3^, ^4^, ^5^, ^6^, ^7^, ^8^, ^9^, ^10^ the factors that drive BNP change after AS correction by TAVR remain unclear.

In the present study, we aimed to assess the magnitude of longitudinal change of BNP in severe AS patients treated by TAVR, its impact on survival, and its association with functional status and underlying LV mechanics. We also assessed the determinants of BNP change and its association with specific LV remodeling patterns.

## 2 METHODS

### 2.1 Study sample

We reviewed our series of 237 consecutive high risk or surgically inoperable patients with severe AS who underwent TAVR through transfemoral access with the Edwards SAPIEN valve at Cleveland Clinic between May 2006 and December 11, 2012.^11^ This included patients who enrolled in clinical trials (ie, REVIVAL, PARTNER trial, and PARTNER II) and patients who underwent commercial TAVR. Plasma BNP measurements and echocardiographic assessments were done in a prospectively determined sequence at baseline, before discharge, 1 month, 6 months, 1 year, and annually thereafter. Baseline and subsequent echocardiographic studies were used to assess the association between BNP change and cardiac reverse remodeling after TAVR. We also evaluated the New York Heart Association (NYHA) functional class and 6‐minute walk test (6MWT) results at the same point. The survival status of all patients after TAVR was collected from medical records or publicly available online sources (last queried April 2017). Patients were followed through chart review with either the date of last follow‐up or the date of death recorded. We considered all‐cause mortality to be the primary outcome. The secondary end point was the rate of death from cardiovascular causes. Cardiovascular deaths were defined as deaths resulting from a cardiac cause including sudden unexplained deaths in which a cardiac cause could not be excluded.^12^ The study protocol was approved by the Cleveland Clinic Institutional Review Board, patient informed consent was waived and data were deidentified.

### 2.2 Echocardiographic measurement

Baseline and subsequent echocardiographic measurements were systematically reviewed and measured by an experienced reader according to the current guidelines blinded to clinical, other variables, and outcome.^13^, ^14^ Measured echocardiographic parameters included LV end‐diastolic volume (LVEDV), LV end‐systolic volume (LVESV), LV ejection fraction (EF), global longitudinal strain (GLS), aortic valve area (AVA), and peak velocity of trans‐aortic valve flow. The LVEDV, LVESV, and LVEF were measured by the biplane Simpson's method from apical views. The AVA was estimated by the 2D‐Doppler method using the continuity equation. The severity of aortic regurgitation (AR) after TAVR was assessed by transthoracic echocardiography within 30 days of the procedure.

**Figure 1:**
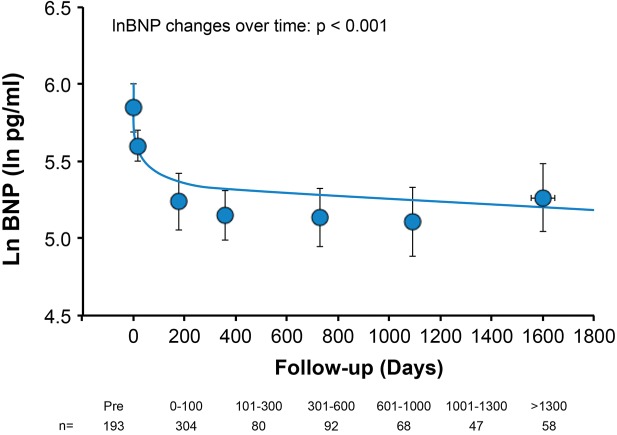
Plasma BNP levels during follow‐up. Markers represent the average of the observed data obtained before TAVR (time zero) over the intervals of 0 to 100 days, 101 to 300 days, 301 to 600 days, 601 to 1000 days, 1001 to 1300 days, and > 1300 days after TAVR. Error bars represent 95% confidence intervals. The regression line was obtained by the mixed model to represent the regression of BNP in all patients. *P*‐values for time after TAVR are shown. Many measurements at each time point were shown in the bottom. BNP, B‐type natriuretic peptide; TAVR, transcatheter aortic valve replacement

LV remodeling was classified according to the combination of presence of LV hypertrophy (LVH) and relative wall thickness (RWT) values as concentric hypertrophy (CH), eccentric hypertrophy (EH), concentric remodeling (CR), and normal (Figure 1, Supporting Information).^13^, ^15^ LV mass index (LVMi) was calculated as described,^13^ with LVH was defined as LVMi > 95 g/m^2^ in female and LVMi > 115 g/m^2^ in male. RWT was calculated as: RWT = 2 × PWT/LVID. CH was diagnosed if patient showed significant LVH and increased RWT (>0.42). While if RWT was normal, patient with LVH was classified as EH. CR was classified when patients with no LVH showed increased RWT.

GLS was assessed by two‐dimensional speckle‐tracking echocardiography as previously reported.^11^ The peak systolic strain values from apical four‐chamber, two‐chamber, and long‐axis views were averaged to obtain GLS.

### 2.3 Functional capacity

In the 156 patients who were enrolled in PARTNER trial and PARTNER II trial, 6MWT was conducted as part of prospectively determined protocol at baseline, 30 days, 6 months, and 1 year after TAVR. In nine patients, the 6MWT assessment was missed at the baseline and these patients were excluded from the analysis. Of note, in 49 patients, 6MWT was scheduled but not completed because of medical reasons (ie, postural hypertension, postural change in heart rate, low resting systolic pressure, patient being non‐ambulatory because of medical condition, such as presence of angina or respiratory insufficiency at rest) and they were coded as zero meters. Data were obtained from research database.

### 2.4 Statistical analysis

Continuous data are expressed as mean ± SD when normally distributed, or median (interquartile range). Categorical data are presented as an absolute number and percentages. BNP values were logarithmically transformed prior to analysis. We used the unpaired *t* test, Mann‐Whitney test, One‐way anova, and Kruskal‐Wallis test to compare the data between two or more groups as appropriate. To assess changes in BNP after TAVR over time, and possible impact of LV remodeling pattern, we applied longitudinal data analysis using a mixed effect model with unstructured covariance for random and fixed effects,^16^, ^17^ and with individual patients treated as random effects. Both BNP and time were logarithmically transformed.^11^ Model selection was accomplished using log‐likelihood ratio testing. We also used a mixed effect model to assess the relationship between BNP levels and LVEDVi or 6MWT. To assess impact of time‐varying effects on BNP survival after TAVR, we constructed a Cox proportional hazards model using BNP levels after TAVR as a segmented time‐dependent covariate as described before.^18^ Serial BNP levels were adjusted for difference in each time period (0, 1‐10 days, 11‐180 days, 181‐450 days, >450 days) and added into the model as a time‐dependent covariate. After constructing initial univariable model with BNP as a time‐dependent covariate, we constructed the multivariable model by forcing baseline BNP and STS score. These variables were selected because of their known prognostic value in AS.^19^ The assumption of proportional hazards were assessed based on the Schoenfeld residuals. A *P*‐value of <0.05 was considered statistically significant. All statistical analyses were performed with JMP 10.0 (SAS Institute Inc., Cary, North Carolina), SPSS 23.0 software (SPSS Inc., Chicago, Illinois), and R software version 3.2.2 (R Foundation for Statistical Computing, Vienna, Austria).

## 3 RESULTS

We identified 193 patients who fulfilled inclusion criteria (Table S1). Six patients were excluded because of lack of baseline BNP assessment while 38 patients were excluded as they were followed by N‐terminal pro BNP levels. “Commercial” TAVR was performed in 29 patients. A total of 30 patients (16%) were classified as having EH, 115 patients (60%) had CH, 37 patients (19%) had CR, and 11 patients (6%) were found to have normal LV geometry. Baseline EF and GLS were significantly reduced in patients with EH compared to patients with CH, CR, and normal patients (Table 1).

**Table 1:** Comparison of laboratory and echocardiographic variables according to remodeling pattern

N = 193	Normal N = 11	CR N = 37	CH N = 115	EH N = 30	*P*‐value
Age (years)	79 ± 13	78 ± 13	82 ± 8	79 ± 10	0.18
Male, n (%)	10 (91%)	24 (65%)	59 (51%)	21 (70%)	0.024
Systolic blood pressure (mm Hg)	127 ± 25	131 ± 20	132 ± 22	117 ± 15	0.020
Body surface area (m^2^)	2.11 ± 0.25	1.93 ± 0.25	1.86 ± 0.28	1.94 ± 0.21	0.008
NYHA ≥ III, n (%)	11 (100%)	34 (92%)	106 (92%)	30 (100%)	0.55
Coronary artery disease, n (%)	9 (82%)	31 (84%)	96 (83%)	26 (87%)	0.97
STS score (risk of mortality)	7.8 ± 3.4	7.8 ± 4.6	10.1 ± 5.2	11.2 ± 5.0	0.016
Creatinine (mg/dL)	1.16 ± 0.33	1.10 ± 0.35	1.17 ± 0.52	1.35 ± 0.52	0.22
BNP (pg/mL)	232 (125‐620)	192 (124‐352)	351 (144‐615)	539 (332‐1515)	0.025
LVEDV index (mL/m^2^)	58 ± 20	47 ± 14	55 ± 19	82 ± 27	<0.001
LVESV index (mL/m^2^)	31 ± 15	19 ± 10	27 ± 16	52 ± 26	<0.001
LVEF (%)	49 ± 10	56 ± 12	52 ± 13	35 ± 13	<0.001
LV mass index (g/m^2^)	89 ± 15	90 ± 16	145 ± 31	154 ± 33	<0.001
Relative wall thickness	0.37 ± 0.04	0.57 ± 0.14	0.61 ± 0.14	0.35 ± 0.05	<0.001
GLS (%	−13 ± 2	−13 ± 3	−12 ± 3	−9 ± 3	<0.001
AV mean PG (mm Hg)	47 ± 17	43 ± 8	49 ± 17	40 ± 13	0.011
AV peak velocity (m/s)	4.41 ± 1.03	4.20 ± 0.39	4.52 ± 0.80	4.06 ± 0.66	0.015
AVA (cm^2^)	0.65 ± 0.20	0.66 ± 0.14	0.61 ± 0.13	0.63 ± 0.20	0.37
AR ≥ moderate	2 (22%)	6 (19%)	17 (18%)	5 (18%)	0.69
30 days					
BNP (pg/mL)	309 ± 324	423 ± 958	373 ± 334	740 ± 760	0.036
Post‐TAVR AR ≥ moderate	1 (8%)	3 (8%)	14 (12%)	4 (13%)	0.81
1 year after					
BNP (pg/mL)	236 ± 227	238 ± 216	243 ± 165	301 ± 181	0.72

Abbreviations: AR, aortic regurgitation; AV, aortic valve; AVA = aortic valve area; BNP,B‐type natriuretic peptide; CH, concentric hypertrophy; CR, concentric remodeling; EH, eccentric hypertrophy; GLS, global longitudinal strain; LV, left ventricle; LVEDV, left ventricular end‐diastolic volume; LVEF, left ventricular ejection fraction; LVESV, left ventricular end‐systolic volume; NYHA, New York Heart Association; STS, Society of Thoracic Surgeons; TAVR, transcatheter aortic valve replacement.

Values are mean ± SD, median (interquartile range), or n (%).

### 3.1 Changes of BNP post‐TAVR

After TAVR, median BNP decreased from 318 (153‐601) to 262 (144‐461) pg/mL in the first 30 days in the entire group with further improvement during follow‐up (182 [116‐338] at 1 year, *P* < 0.001) (Figure 1).

In a next step, we divided patients into three groups according to their survival: patients who died during first 450 days, patients who died between 450 and 1200 days, and patients who survived for more than 1200 days. All three groups experienced a similar drop in BNP after TAVR (interaction between groups with time: *P* = 0.07). This indicates that decrease in BNP during follow‐up was not related to the informative censoring (ie, caused by elimination through earlier death of patients with high BNP, which then eliminates them from contributing to BNP values at later follow‐up times).

### 3.2 Impact of LV remodeling pattern on BNP change after TAVR

**Figure 2:**
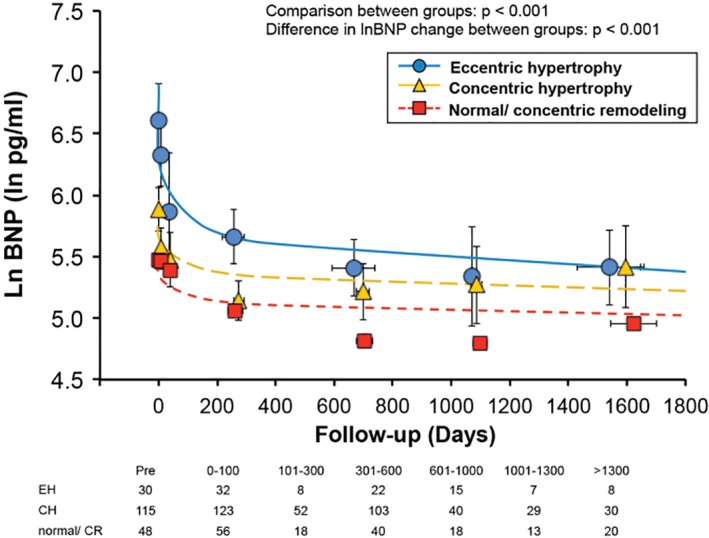
Plasma BNP levels according to remodeling pattern. See Figure 1 legend for details. The significant *P‐*values for comparison between groups, change over time, and interaction between group and change are shown. BNP, B‐type natriuretic peptide; CH, concentric hypertrophy; CR, concentric remodeling; EH, eccentric hypertrophy

Figure 2 shows changes of BNP levels according to LV remodeling patterns. BNP levels were higher in patients with EH at baseline (*P* < 0.001). There was interaction between LV remodeling pattern and time, suggesting that patients with EH demonstrated a greater subsequent decrease in BNP levels after TAVR compared to those with CH, CR, and normal patterns (EH: −717 vs CH: −178 vs CR/ normal: −99 pg/mL change at 1 year, *P* < 0.001 for interaction between remodeling pattern with time). While significant drop in BNP was observed immediately after TAVR but continued through 6 months to 1 year after TAVR. When we compare the %BNP change according to the remodeling pattern after TAVR, EH group showed greatest %BNP decrease at 100 to 300 days after TAVR and continued with slow decrease afterwards (Supplemental Figure 2).

### 3.3 Factors associated with BNP levels after TAVR

**Figure 3:**
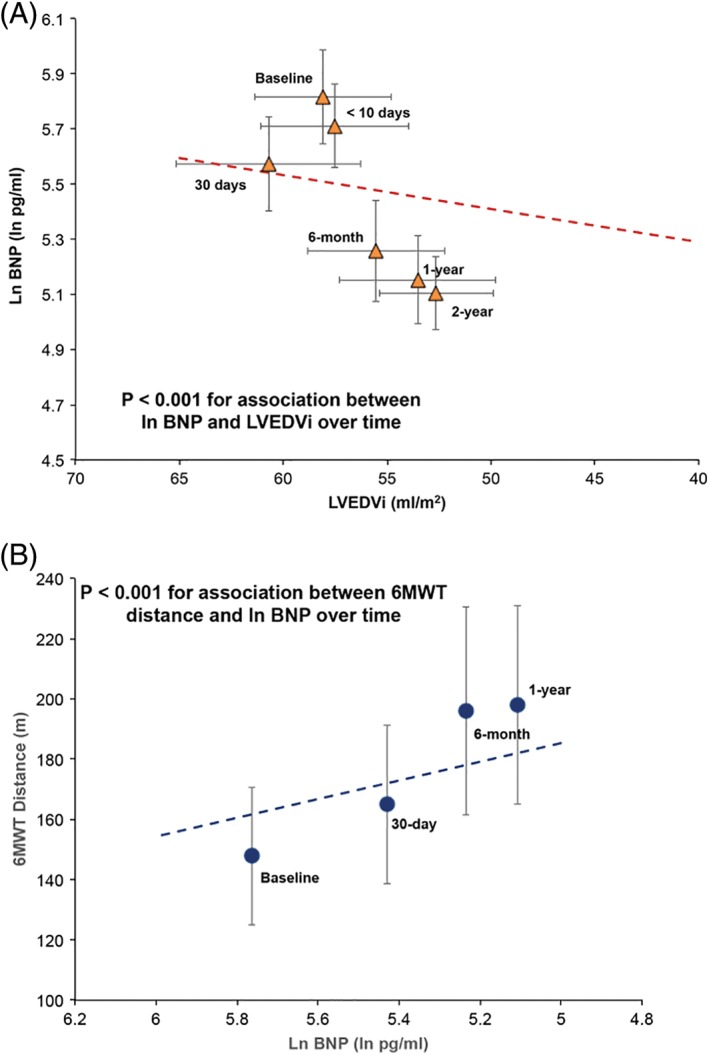
A, Association between BNP and LVEDVi during follow‐up. BNP and LVEDVi decreased in parallel throughout follow‐up (*P* < 0.001 for the association; see text for details). B, Association between BNP and 6‐minute walk test during follow‐up. A decrease in BNP paralleled improvement in 6‐minute walk test distance during follow‐up (*P* < 0.001 for association; see text for details). Ln BNP, natural logarithm of B‐type natriuretic peptide concentration; LVEDVi, left ventricular end‐diastolic volume index; 6MWT, 6‐minute walk test distance

BNP levels were associated with LVEDVi over the follow‐up period (*P* < 0.001, Figure 3A). We also show a parallel decrease of BNP and LVEDVi during follow‐up, suggesting that changes in LVEDVi and in BNP after TAVR coincide without significant time lag between the two. There was significant association between BNP improvement and GLS over time after TAVR (*P* < 0.001) (Supplemental Figure 3). Suggesting improvement in LV contractility is coincide with BNP change.

To assess the association between functional class and BNP levels, we collected NYHA class and 6MWT results. At baseline, 49 patients were unable to walk because of medical conditions and 6MWT distance was considered as 0 m. After TAVR, among those 49 patients, 19 patients were able to walk. The mean distance of the 6MWT was 222 ± 116 m at baseline and improved to 264 ± 121 m at 30 days after TAVR, followed by further subsequent improvement to 271 ± 124 m at 6 months after TAVR (*P* = 0.010) (Table 2). In a mixed effect model, BNP levels showed significant negative correlation with 6MWT distance at baseline and over the follow‐up period. Significant parallel improvement in 6MWT distance and ln BNP level were observed (*P* < 0.001, Figure 3B). Similarly, NYHA class was significantly improved from 3.0 ± 0.4 to 2.1 ± 0.6 at 30 days after TAVR and also showed further improvement to 1.6 ± 0.7 at 1 year after TAVR (*P* < 0.001).

**Table 2:** Changes of lnBNP, 6‐minute walk test distance, and NYHA after TAVR

Follow‐up duration (days)	Baseline	30 days	6 months	1 year	2 year
Ln BNP (pg/mL)	5.8 ± 1.0	5.4 ± 0.8	5.2 ± 0.8	5.1 ± 0.8	5.1 ± 0.8
6MWT distance (m)	222 ± 116	264 ± 121	271 ± 124	278 ± 135	—
NYHA class	3.0 ± 0.4	2.1 ± 0.6	1.8 ± 0.7	1.6 ± 0.7	1.7 ± 0.7

Abbreviations: 6MWT, 6‐minute walk test; lnBNP, natural logarithm of B‐type natriuretic peptide concentration; NYHA, New York Heart Association; TAVR, transcatheter aortic valve replacement.

### 3.4 Prognostic impact of BNP levels after TAVR

During a median follow‐up period of 1331 days (interquartile range: 632‐1678), 119 (62%) patients died of any cause during follow‐up, with 48 patients (25%) dying during first 2 years. Of those 119 patients who died during follow‐up, 81 died because of cardiac cause. Furthermore, 74 patients died at 5 years of follow‐up, and the risk of cardiovascular‐related mortality at 5 years was 38%. To explore the impact of changing BNP levels during follow‐up, we performed uni‐ and multivariable Cox proportional hazards model with post‐TAVR BNP levels as a segmental time‐dependent covariate. In a univariable model, BNP as a time‐dependent covariate was a significant predictor of survival (hazards ratio [HR] 1.24, 95% CI: 1.04‐1.47, *P* = 0.017). In a multivariable model, BNP as a time‐dependent covariate remained significant after adjusting for STS score (HR 1.20, 95% CI: 1.01‐1.44, *P* = 0.041), and baseline BNP levels (HR 1.33, 95% CI: 1.01‐1.73, *P* = 0.040), or both (HR 1.32, 95% CI: 1.001‐0.73, *P* = 0.049). Kaplan‐Meier curves show that patients with high BNP (>400 pg/mL) at 1 year post‐TAVR had poorer survival after TAVR (Log‐rank *P* = 0.03) (Figure S4). These data suggest the importance of ongoing surveillance with BNP level after TAVR to predict their survival.

Moreover, when we repeated the analysis after excluding patients with EH, BNP was still a significant predictor of death with HR of 1.40 (95% CI: 1.02‐1.90, *P* = 0.036). On the other hand, BNP was not significantly associated with survival in patients with EH (HR 1.16, 95% CI: 0.62‐2.14, *P* = 0.65).

## 4 DISCUSSION

In the present study, we explored the interconnection between processes of reverse remodeling, improvement in BNP levels, and increased exercise capacity that concomitantly occur after TAVR. Overall, we observed that after TAVR patients with adverse LV remodeling show a continuing decrease in BNP during follow‐up. This improvement in BNP coincided with recovery of cardiac structure and function. However, improvement in BNP was much less noticeable in patients without eccentric remodeling. The 6MWT distance, a marker of exercise capacity, also improved after TAVR, with ability to walk a longer distance again coinciding with lower BNP levels. The time‐dependent changes in post‐procedural BNP levels were associated with all‐cause mortality after TAVR.

### 4.1 Association of BNP levels and LV remodeling pattern after TAVR

As it is known that BNP release is mediated by increased diastolic myocardial wall stress,^20^, ^21^ monitoring of BNP over time is frequently used as a noninvasive estimation of LV filling pressures. What happens with LV end‐diastolic pressure during follow‐up is difficult to ascertain, as it would necessitate repeated cardiac catheterization. In this setting, BNP could be viewed as a surrogate marker of LV filling pressures. In addition, elevated BNP is linked to other high‐risk features, such as aging and renal disease, and thus tracks well with the development and severity of heart failure symptoms in AS.^2^, ^22^ Hence, we postulated that elevated BNP levels might be a surrogate for maladaptive transition in LV remodeling.^1^ Our study confirmed these findings by showing that EH patients had higher baseline BNP levels.^23^ We also show that, despite worse baseline LV structural and functional characteristics and higher BNP levels in patients with EH, these patients show a largest decrease in BNP levels following TAVR, and that BNP decrease closely follows normalization of LVEDVi. Finally, while prior studies showed reversal of BNP levels was incomplete in most patients at 1 year^6^, we show that improvement continues during 5‐year follow‐up. Limited and slow recovery in BNP is probably because regression of LVH and improvement in scar content are slow processes and may require years to complete.^24^

### 4.2 Functional improvement, BNP levels, and survival after TAVR

As elevated LV filling pressures and diastolic wall stress are factors causing both decreased functional capacity and elevated BNP levels, one might expect that, mechanistically, BNP levels should correlate with exercise capacity. Indeed, BNP tracks changes of functional capacity in patients with chronic systolic heart failure.^25^ However, the relationship between exercise capacity and BNP following correction of valve disease in elderly patients is less clear. TAVR patients are often frail, but with preserved LVEF. The 6MWT has been used as a maker of frailty.^26^ It also appears that the 6MWT is a useful surrogate of maximum exercise capacity in patients with heart failure and preserved ejection fraction.^27^ Finally, recent data show that improvement in the 6MWT following TAVR is associated with better survival..^28^ We add to these findings by showing that post‐TAVR, a single measure of 6MWT is not necessarily sufficient as it changes over time, while closely following BNP. Finally, we show that changing BNP levels are associated with survival after TAVR.

It appears to be that restoration of increased LV wall stress leads LV reverse remodeling, such as reduction of LV volume or improvement of systolic function, improvement in functional capacity, and BNP levels. This recovery was also associated with favorable prognosis after TAVR. In sum our findings show that TAVR leads to complex dynamic changes in cardiac structure, BNP levels and exercise capacity that eventually result in improved survival.

### 4.3 Clinical implication

In the present paper, we demonstrate that BNP levels after TAVR reflect the dynamic changes in cardiac structure and function. We first show that TAVR leads to early drop in BNP. We also show that there is a further continuing fall in BNP levels after TAVR, which is associated with improved LV structure and functional capacity. On the other side, we also show that a subsequent increase in BNP during follow‐up is associated with increased chance of all‐cause mortality. These findings suggest that BNP provides a mechanistic link between LV remodeling pattern and improved functional capacity in AS patients. Hence, elevated BNP can serve as a surrogate of unfavorable clinical outcome after TAVR. BNP may be an inexpensive, convenient marker of post‐TAVR cardiac remodeling, exercise capacity, and survival.

### 4.4 Limitations

This was a retrospective observational study conducted at a large tertiary referral center and thus may suffer from selection bias. In the present study, only Edwards SAPIEN and SAPIEN XT valves were used, and the majority of patients underwent TAVR with the first generation SAPIEN valve. Moreover, although data were collected prospectively and analyzed by a mixed effect model, half of the patients died during the first 5 years of follow‐up, which may result in survivorship bias.

## 5 CONCLUSIONS

BNP levels were associated with a LV remodeling phenotype and functional status pre‐ and post‐TAVR. Regression of BNP levels was prominent in patients with EH. Decrease of BNP levels was also associated with improved functional capacity. Elevated BNP levels after TAVR were independently associated with poor prognosis. In patients who underwent TAVR, serial follow‐up of plasma BNP levels is useful in estimating prognosis.

## CONFLICTS OF INTEREST

The authors declare no potential conflict of interests.

## Supporting information


**FIGURE S1** Left ventricular remodeling pattern.

**FIGURE S2** Relative change of BNP when we stratified patients according to remodeling pattern. Markers represent the average of the observed data obtained before TAVR (time zero) over the intervals of 0 to 100 days, 101 to 300 days, 301 to 600 days, 601 to 1000 days, 1001 to 1300 days, and > 1300 days. Error bars represent 95% confidence intervals. BNP, B‐type natriuretic peptide; CH, concentric hypertrophy; CR, concentric remodeling; EH, eccentric hypertrophy.

**FIGURE S3** Association between BNP and GLS during follow‐up. BNP and GLS decreased in parallel throughout follow‐up (*P* < 0.001 for the association). Ln BNP, natural logarithm of B‐type natriuretic peptide concentration; GLS, global longitudinal strain; TAVR, transcatheter aortic valve replacement.

**FIGURE S4** Kaplan‐Meier curves according to BNP level at 1‐year after TAVR. Survival curves showed patients with high plasma BNP level at 1 year after TAVR associated with higher mortality (Log‐rank *P* < 0.001). BNP, B‐type natriuretic peptide; TAVR, transcatheter aortic valve replacement.

**TABLE S1** Patient clinical characteristics
