# Supplementary figures and images for: B‐type natriuretic peptide is associated with remodeling and exercise capacity after transcatheter aortic valve replacement for aortic stenosis
Source: Clin Cardiol. 2018 Dec 31;42(2):270–6. doi: 10.1002/clc.23138 (PMC6712326; doi:10.1002/clc.23138)

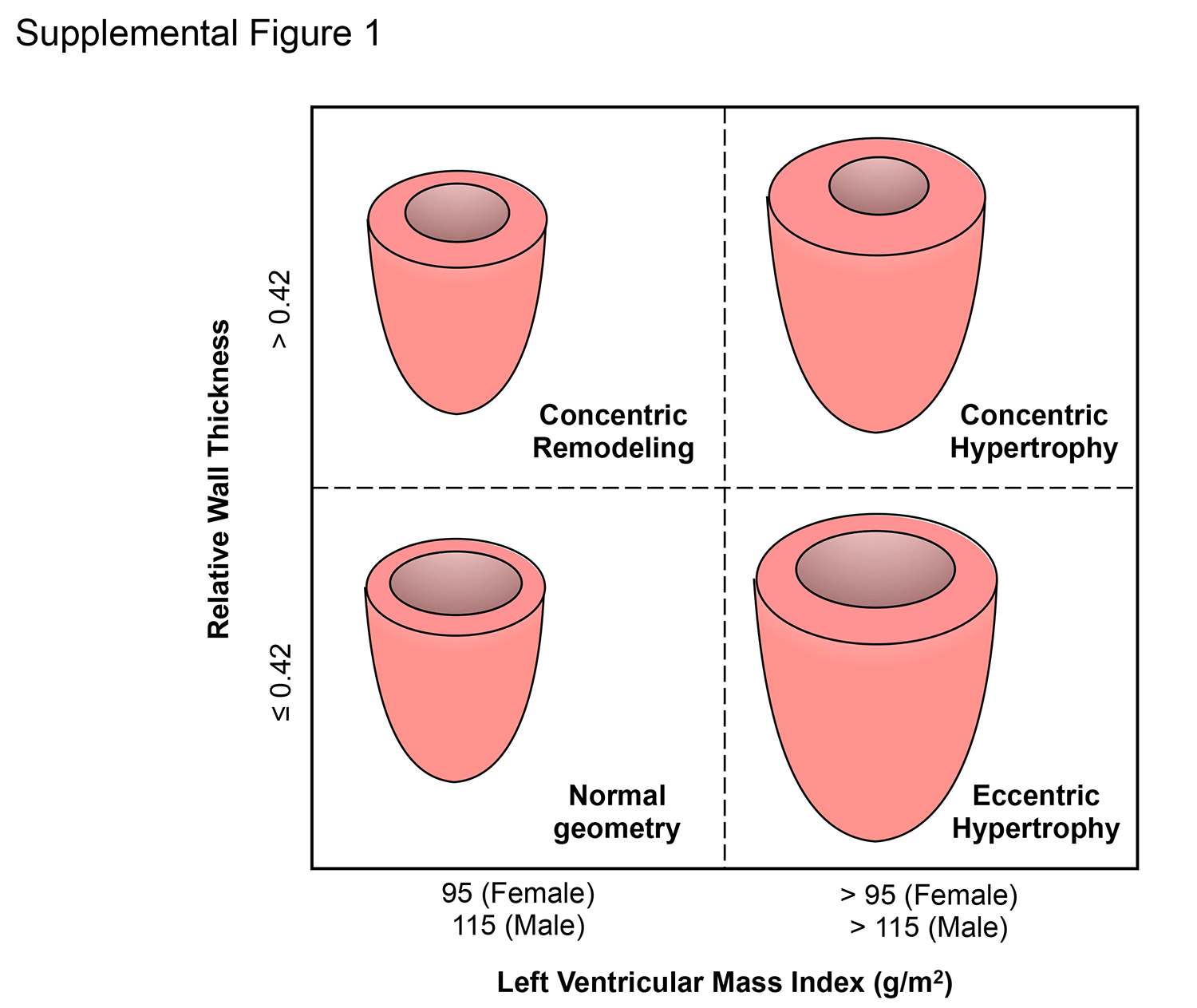

Supplement: Supplementary file 1 — FIGURE S1 Left ventricular remodeling pattern. [file CLC-42-270-s005.tif]

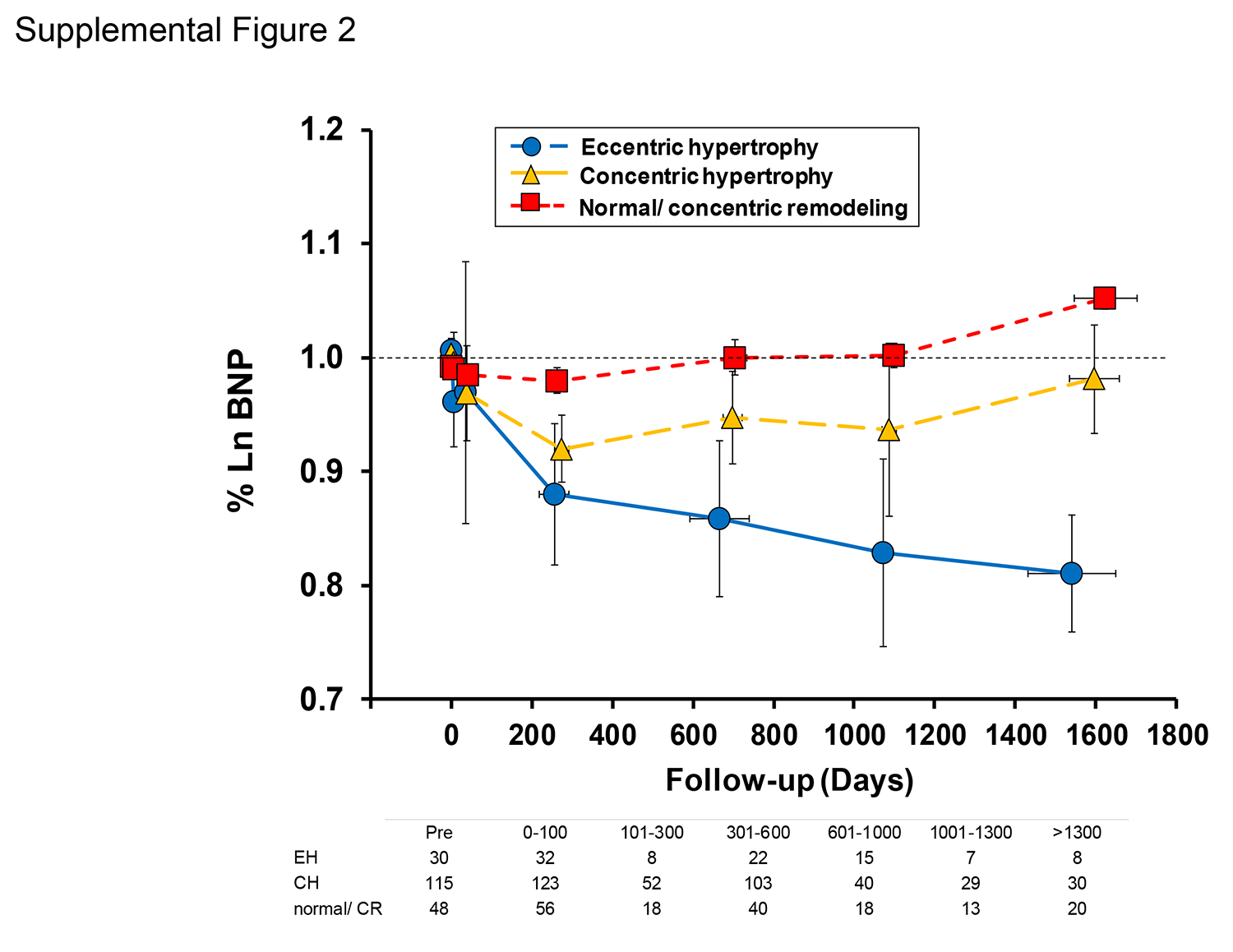

Supplement: Supplementary file 2 — FIGURE S2 Relative change of BNP when we stratified patients according to remodeling pattern. Markers represent the average of the observed data obtained before TAVR (time zero) over the intervals of 0 to 100 days, 101 to 300 days, 301 to 600 days, 601 to 1000 days, 1001 to 1300 days, and > 1300 days. Error bars represent 95% confidence intervals. BNP, B‐type natriuretic peptide; CH, concentric hypertrophy; CR, concentric remodeling; EH, eccentric hypertrophy. [file CLC-42-270-s004.tif]

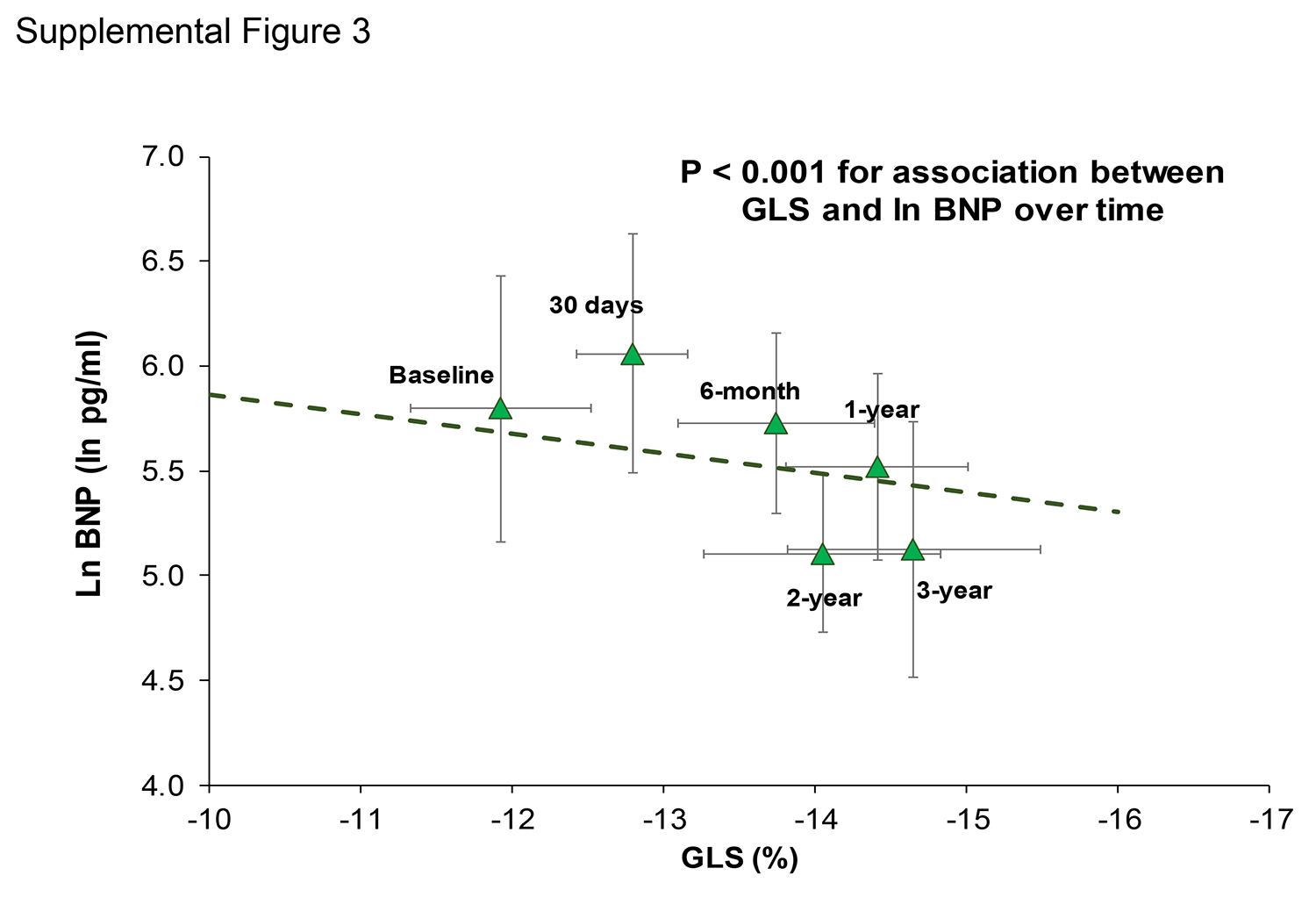

Supplement: Supplementary file 3 — FIGURE S3 Association between BNP and GLS during follow‐up. BNP and GLS decreased in parallel throughout follow‐up (P < 0.001 for the association). Ln BNP, natural logarithm of B‐type natriuretic peptide concentration; GLS, global longitudinal strain; TAVR, transcatheter aortic valve replacement. [file CLC-42-270-s003.tif]

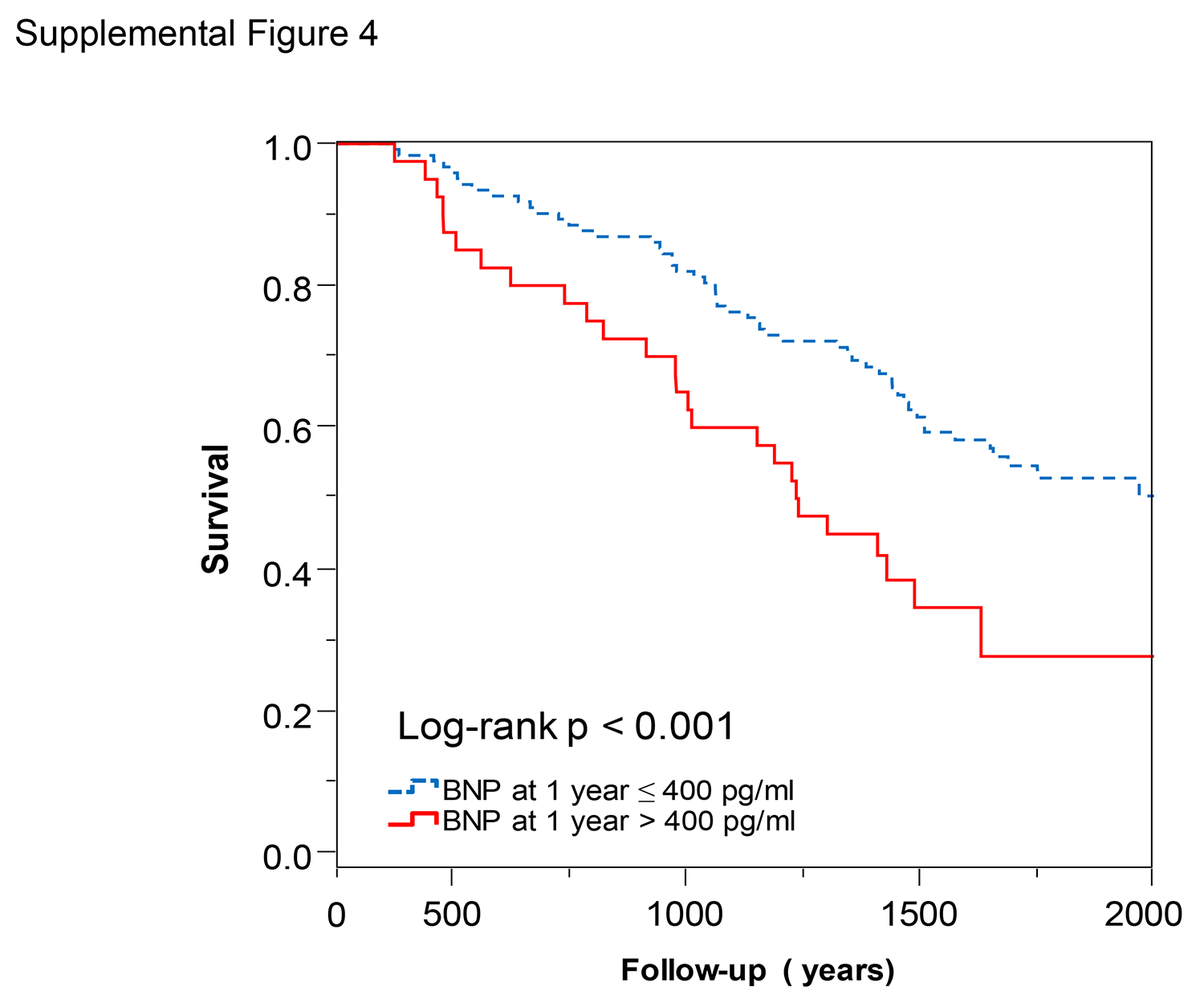

Supplement: Supplementary file 4 — FIGURE S4 Kaplan‐Meier curves according to BNP level at 1‐year after TAVR. Survival curves showed patients with high plasma BNP level at 1 year after TAVR associated with higher mortality (Log‐rank P < 0.001). BNP, B‐type natriuretic peptide; TAVR, transcatheter aortic valve replacement. [file CLC-42-270-s002.tif]
