# Supplementary material for: B‐type natriuretic peptide is associated with remodeling and exercise capacity after transcatheter aortic valve replacement for aortic stenosis
Source: Clin Cardiol. 2018 Dec 31;42(2):270–6. doi: 10.1002/clc.23138 (PMC6712326; doi:10.1002/clc.23138)
Supplement: Supplementary file 5 — TABLE S1 Patient clinical characteristics [file CLC-42-270-s001.docx]

**SUPPLEMENTAL MATERIALS**

**Supplemental Table**

**Supplemental Table 1. Patient Clinical Characteristics**

|  | **All patients (n = 193)** |
| --- | --- |
| **Age, years** | 80 ± 10 |
| **Male, n (%)** | 114 (59%) |
| **Systolic blood pressure, mmHg** | 129 ± 21 |
| **Body surface area, m^2^** | 1.90 ± 0.27 |
| **NYHA ≥ III, n (%)** | 181 (94%) |
| **Coronary artery disease, n (%)** | 162 (84%) |
| **STS score (risk of mortality), %** | 9.7 ± 5.1 |
| **Creatinine (mg/dl)** | 1.18 ± 0.48 |
| **First generation valve, n (%)** | 173 (92%) |

Values are mean ± SD, median (interquartile range), or n (%).

Abbreviations: NYHA = New York Heart Association; STS = Society of Thoracic Surgeons.

**Supplemental Figure Legends**

**Supplemental Figure 1. Left ventricular remodeling pattern.**

**Supplemental Figure 2. Relative change of BNP when we stratified patients according to remodeling pattern.** Markers represent the average of the observed data obtained before TAVR (time zero) over the intervals of 0 to 100 days, 101 to 300 days, 301 to 600 days, 601 to 1,000 days, 1,001 to 1,300 days, and > 1,300 days. Error bars represent 95% confidence intervals.

Abbreviations: BNP = B-type natriuretic peptide; CH = concentric hypertrophy; CR = concentric remodeling; EH = eccentric hypertrophy.

**Supplemental Figure 3.** **Association between BNP and GLS during follow-up.**

BNP and GLS decreased in parallel throughout follow up (p < 0.001 for the association).

Abbreviations: Ln BNP = natural logarithm of B-type natriuretic peptide concentration; GLS = global longitudinal strain; TAVR = transcatheter aortic valve replacement.

**Supplemental Figure 4. Kaplan Meier curves according to BNP level at 1-year after TAVR.** Survival curves showed patients with high plasma BNP level at 1 year after TAVR associated with higher mortality (Log-rank p < 0.001)

Abbreviations: BNP = B-type natriuretic peptide; TAVR = transcatheter aortic valve replacement.
